# Unraveling the politics of ‘doing inclusion’ in transdisciplinarity for sustainable transformation

**DOI:** 10.1007/s11625-021-01033-7

**Published:** 2021-09-13

**Authors:** Kristiaan P. W. Kok, Mads D. Gjefsen, Barbara J. Regeer, Jacqueline E. W. Broerse

**Affiliations:** 1 Athena Institute, VU University Amsterdam, De Boelelaan 1085, 1081 HV Amsterdam, The Netherlands; 2 Work Research Institute, OsloMet – Oslo Metropolitan University, St. Olavs plass, Postboks 4, 0130 Oslo, Norway

**Keywords:** Transdisciplinarity, Transformation, Stakeholder inclusion, Power, Living Labs, Sustainability transitions

## Abstract

Transdisciplinary research and innovation (R&I) efforts have emerged as a means to address challenges to sustainable transformation. One of the main elements of transdisciplinary efforts is the ‘inclusion’ of different stakeholders, values and perspectives in participatory R&I processes. In practice, however, ‘doing inclusion’ raises a number of challenges. In this article, we aim to contribute to re-politicizing inclusion in transdisciplinarity for transformation, by (1) empirically unraveling four key challenges that emerge in the political practice of ‘doing inclusion’, (2) illustrating how facilitators of inclusion processes perform balancing acts when confronted with these challenges, and (3) reflecting on what the unfolding dynamics suggests about the politics of stakeholder inclusion for societal transformation. In doing so, we analyze the transdisciplinary FIT4FOOD2030 project (2017–2020)—an EU-funded project that aimed to contribute to fostering EU R&I systems’ ability to catalyze food system transformation through stakeholder engagement in 25 Living Labs. Based on 3 years of action-research (including interviews, workshops and field observations), we identified four inherent political challenges to ‘doing inclusion’ in FIT4FOOD2030: (1) the challenge to meaningfully bring together powerful and marginalized stakeholders; (2) combining representation and deliberation of different stakeholder groups; (3) balancing diversities of inclusion with directionalities implied by transformative efforts; and (4) navigating the complexities of establishing boundaries of inclusion processes. We argue that by understanding ‘doing inclusion’ as a political practice, necessitating specificity about the (normative) ambitions in different inclusion settings, facilitators may better grasp and address challenges in transdisciplinarity for transformation.

## Introduction

Research and innovation (R&I) processes can help foster urgently needed sustainable and just transformations in socio-ecological and socio-technical systems (Fazey et al. 2018, 2020; Norström et al. 2020; West et al. 2020). *Transdisciplinary approaches* show particular promise by including societal stakeholders in research, innovation and governance efforts (Miller et al. 2014; Lang and Wiek 2021). Various inclusive R&I approaches aim to bridge the gap between ‘knowledge and action’ (van Kerkhoff and Lebel 2006; West et al. 2019), including Transition Management (Loorbach 2007), Responsible Research and Innovation (RRI, see Owen et al. 2012), transformative research (Fazey et al. 2018) and transdisciplinarity (Klein et al. 2001; Lang et al. 2012). Though different in approach and underlying philosophies, these approaches share deep commonalities, among them the notion that problem-driven, iterative R&I efforts could—more effectively than traditional linear processes—contribute to tackling societal challenges by co-producing knowledge with researchers and societal stakeholders through processes that acknowledge diversity of knowledges and values while fostering learning and reflexivity among participating actors (Lang et al. 2012; Caniglia et al. 2020; Lang and Wiek 2021).

Undervaluing the intrinsic political nature of ‘doing inclusion’ risks losing sight of how the politics of participation drives the dynamics of transdisciplinary processes (Chilvers and Kearnes 2020; Stirling 2008). However, in a recent review, Turnhout et al. (2020) indicate that the political dynamics of transdisciplinary processes aimed at transformation often remain underemphasized in both practice and research, and most scholarship tends to focus on addressing and enacting practical, methodological or institutional aspects of transdisciplinarity (such as in Pohl and Hadorn 2008; Lang et al. 2012; Brandt et al. 2013). This focus seems likely to intensify within the recently observed turn towards effectiveness-orientation in, and functionalization of, stakeholder inclusion in transdisciplinarity (Musch and von Streit 2020; Chilvers and Kearnes 2020; Schmidt et al. 2020). This emphasis on ‘effectiveness’ in transdisciplinarity arises in part due to the *“trend that funding agencies increasingly favour transdisciplinary projects focusing on directly applicable outputs”* (Musch and von Streit 2020: 63). Stakeholder inclusion might devolve into ‘tick-the-box’ requirements, or worse: lead to tokenism or oppression through participation (e.g., Cooke and Kothrari 2001). This functional turn is rather surprising as other rationales for doing stakeholder inclusion, such as promoting social learning and reflexivity, enhancing legitimacy of R&I processes and outcomes, as well as efforts for democratizing R&I in response to socially unjust outcomes, lie at the very core of transdisciplinarity (see, e.g., Jasanoff 2003; van Kerkhoff and Lebel 2006; Brown 2009; Bunders et al. 2010; Schmidt et al. 2020). Critiques of the functional turn (see Chilvers and Kearnes 2020) also led scholars to argue that there is a need *“for a new phase of ‘democratization of science’”* (Cornell et al. 2013: 68) that entails a thorough *“rethinking and a repoliticization”* (Turnhout et al. 2020: 18) of inclusion for transformation.

In this article, we aim to contribute to re-politicizing inclusion in transdisciplinarity for transformation, by (1) empirically unraveling four key challenges that emerge in the political practice of ‘doing inclusion’, (2) illustrating how facilitators of inclusion processes perform balancing acts when confronted with these challenges, and (3) reflecting on what the unfolding dynamics suggests about the politics of stakeholder inclusion for societal transformation.

Empirically, our puzzle unfolds around ‘doing inclusion’ in the FIT4FOOD2030 project (2017–2020), a Horizon 2020 Coordination and Support Action (CSA) that supported the European Commission (EC) in implementing the FOOD 2030 policy framework. The project’s main goal was to set up a transformative network (including 25 Living Labs on local, regional and national levels) in a move towards *transdisciplinary inclusion* to better enable incumbent R&I systems to facilitate transformations towards sustainable and healthy food systems (see EC 2021; Kok et al. 2019). Before elaborating on our empirical case and analysis, we first set out to further explore the politics of inclusion in transdisciplinary processes aimed at societal transformation.

## The politics of inclusion in transdisciplinarity for transformation

In efforts to contribute to tackling complex and wicked societal challenges (Arkesteijn et al. 2015; Kampelmann et al. 2018, cp. Rittel and Webber 1973), *transdisciplinarity for transformation* seeks to include societal stakeholders in R&I efforts. This section relates complex system transformation to transdisciplinarity, elaborates on different rationales for doing ‘stakeholder inclusion’, and presents key aspects of the politics of inclusion.

### Transdisciplinarity for complex societal transformation

Sustainability transitions are long-term processes of structural systemic change and imply *“far-reaching changes along different dimensions: technological, material, organizational, institutional, political, economic, and socio-cultural”* (Markard et al. 2012: 956). Instigating desired transition pathways (Geels and Schot 2007) or sustainability pathways (Leach et al. 2010) means confronting undesirable resilience (Oliver et al. 2018), incumbency (Stirling 2019), and locked-in equilibrium states (Geels 2002; Grin et al. 2010). In response to such dynamics, scholars have suggested modes of governance (among them Strategic Niche Management, Kemp et al. 1998; Transition Management, see Loorbach 2007) to facilitate processes of experimentation and co-creation. Sengers et al. (2019: 161) conceptualize such processes of experimentation as “*inclusive, practice-based and challenge-led initiative[s] designed to promote system innovation through social learning under conditions of uncertainty and ambiguity*”. Experiments are important as they might serve as protected spaces for building lasting multi-stakeholder networks, co-designing novel solutions and transition pathways, while stimulating learning and reflexivity among participants (Grin et al. 2010; Fazey et al. 2018; Sengers et al. 2019).

Transdisciplinary R&I efforts have emerged in recent decades as a *“new form of learning and problem solving involving cooperation among different parts of society and academia in order to meet complex challenges of society”* (Klein et al. 2001: 7). Inclusive transdisciplinary approaches underlying experimentation and co-creation for sustainable transformation are rapidly gaining ground in academic and policy environments (Fazey et al. 2020; Norström et al. 2020) and form an integral part of transition studies (Grin et al. 2010; Fazey et al. 2018). An overview by Köhler et al. (2019: 19, drawing on Schneidewind et al. 2016; Luederitz et al. 2017; Kampelmann et al. 2018) points to an *“increasing commitment to research that not only describes societal transformation processes, but initiates and catalyzes them”.* One key element in (transformative) transdisciplinarity concerns the *inclusion* of a wide variety of stakeholders from different scientific disciplines as well as societal actors such as policy makers, businesses, civil society and citizens (the Quadruple Helix, see, e.g., Leydesdorff 2012).

### Doing inclusion in transdisciplinarity

Including societal stakeholders in R&I processes is neither a ‘tick-the-box’ activity, nor the panacea for ensuring that R&I processes are democratic, responsible or legitimate (e.g., Cooke and Kothari 2001; Few et al. 2007; Genus and Stirling 2018; Brand and Blok 2019; van Mierlo et al. 2020; Stelzer 2020). Yet, meaningful societal stakeholder engagement can provide ‘better’, more socially robust R&I processes and outcomes (Jasanoff 2003; Bunders et al. 2010; Owen et al. 2012).

In a recent contribution, Schmidt et al. (2020) indicate that literature generally considers four different arguments for doing inclusion. The first is a democratic or normative one, building on, i.e., Arnstein (1969), Fiorino (1990) and Stirling (2008), and stating that those affected by R&I (outcomes) should also have the opportunity to be involved in the process (*‘nothing about us without us!’*). This argument reflects insights on democratic foundations of public deliberation and participation (see Habermas 1981; Dryzek 2002; Collins and Evans 2002; Cash et al. 2003; Nowotny et al. 2003; Jasanoff 2003; Latour 2004). A second argument is a substantive one, namely that R&I that is co-produced between science and society can lead to ‘better’ R&I outcomes. Examples might include more socially robust innovations that are better equipped to provide solutions to real-world challenges, due to the integration of different (stakeholder) perspectives, values and knowledge (Nowotny et al. 2003; Lang et al. 2012). This is especially relevant for designing transformation pathways towards sustainability (Fazey et al. 2018, 2020; Caniglia et al. 2020; West et al. 2020; Den Boer et al. 2021a). A third argument is that transdisciplinary co-production of R&I leads to increased legitimacy of processes and outcomes, especially in the context of implementation of R&I interventions (van Kerkhoff and Lebel 2006; Stirling 2008; Lang et al. 2012). This argument also lies at the core of efforts to make R&I more responsible (for instance in RRI; see von Schomberg 2013; Owen et al. 2012; Stilgoe et al. 2013). Schmidt et al (2020: 3) contend that *“the experience of having had influence on the research process can create a feeling of ownership, increase trust and stimulate commitment among participants in the project and its outcomes”.* The fourth argument concerns social learning and reflection. Bringing together stakeholders from different backgrounds in co-creation processes can stimulate learning, reflexivity and build trust and understanding between participants (Innes and Booher 2004; Hirsch Hadorn et al. 2006; Mathur et al. 2008; Reed et al. 2010; Westberg and Polk 2016). This collective learning is a key element of experimentation for sustainable transformation (Loeber et al. 2007; Grin et al. 2010; Luederitz et al. 2017; van Mierlo and Beers 2020).

While often central to the opening up of R&I processes (Owen et al. 2012), increasing attention is also paid to how ‘inclusion’ relates to processes of exclusion and the (empirical) limits of transdisciplinary efforts (Stirling 2008; de Hoop et al. 2016; Genus and Stirling 2018; Valkenburg et al. 2020; van Mierlo et al. 2020, Koch 2020). Recent scholarship questions whether ‘inclusion’ is always desirable, given the corresponding necessity of processes for closing down (van Mierlo et al. 2020).

### Politics and power in inclusion for transformation

Against the backdrop of the functional turn in participatory approaches, Chilvers and Kearnes (2020) indicate that ‘doing inclusion’ is a deeply political act as it raises the question of who or what decides who is to participate in what way. These questions are also addressed in long-standing debates within *Science and Technology Studies* on deliberative versus representative democratic principles and the role of lay-publics versus experts (see Collins and Evans 2002; Dryzek 2002; Jasanoff 2003; Latour 2004; Meadowcroft 2004; Brown 2009; Turnhout et al. 2010; Chilvers and Longhurst 2016).

It is thus not surprising that scholars point to the role of power (gradients) and agency^1^ in shaping, enhancing, and/or obstructing participatory processes (e.g., Schmidt and Pröpper 2017; Siebenhüner 2018; Bréthaut et al. 2019; Turnhout et al. 2020; Dannecker 2020) and sustainable transformation processes (e.g., Avelino and Rotmans 2009; Grin 2010; Ahlborg 2017; Stirling 2019; Kok et al. 2021; Avelino 2021). If R&I processes are depoliticized or do not address unequal power relations, inclusive (research) efforts risks reproducing incumbent interests and systemic inequities (Cooke and Kothari 2001; Nadasdy 2003, Turnhout et al. 2020). These political dynamics especially matter in the context of transformation, where transdisciplinary processes are not just about providing *“discursive spaces, [but are] attempts to explicitly intervene in system change”* (Chilvers and Longhurst 2016: 587). This in turn relations requires *“finding ways of working with and around the power relations, which shape and are being shaped by the emerging community”* (van Breda and Swilling 2019: 834-835).

What adds to this challenge is the need to both draw upon and *redirect* power relations in building transformative agency within emerging transdisciplinary networks, to contribute to system transformations (see, e.g., Westley et al. 2013; Avelino and Rotmans 2009; Bulkeley et al. 2016; Kok et al. 2021). Such an *interventionist* take on R&I (see also Zuiderent-Jerak 2015; Fazey et al. 2018) raises questions concerning the legitimacy of transdisciplinary processes and the accountability for both transformation processes and outcomes (Hendriks 2008; Hendriks and Grin 2007; Brown 2009). Though ‘inclusion’ could enhance the legitimacy of R&I processes, and lead to shared responsibility and accountability between societal stakeholders and researchers (Nowotny et al. 2003; Lang et al. 2012; von Schomberg 2013; Owen et al. 2012), in messy transdisciplinary practice it is not necessarily clear to whom or what the processes should be accountable (Maasen and Lieven 2006) and on what (democratic) basis accountabilities open up R&I or *“reinforce (rather than fully interrogate) political closures”* (Genus and Stirling 2018: 63, drawing on Chilvers 2008).

## Case: FIT4FOOD2030 as an inclusive instrument for system transformation

In response to the urgent need to set in motion the transformation towards more sustainable and healthier (EU) food systems (e.g., Willett et al. 2019; Rockström et al. 2020), the EC through its Directorate General of Research and Innovation launched the FOOD 2030 policy framework in 2016 (European Commission 2017). The FOOD 2030 policy framework aimed to

*“tackle the [Food and Nutrition Security] challenge with research and innovation (R&I) policies designed to future-proof our food systems to make them sustainable, resilient, diverse, inclusive and competitive for the benefit of society.”* (EC 2017: 4).

To support the EC in delivering FOOD 2030, the FIT4FOOD2030 project was launched in 2017. The transdisciplinary project brought together 16 partner institutions across Europe from research, industry, science communication and civil society, and had the explicit aim of establishing a

*“sustainable, balanced, multi-stakeholder, multi-level platform—called the FOOD2030 Platform—that will support the EC to further develop and implement the FOOD 2030 policy framework and its action plan”* (FIT4FOOD2030 2017: 143).

The project’s main instrument for instigating multi-stakeholder engagement in the transformation of R&I systems was a highly diverse set of 25 Labs. They built on the concept of *Living Labs,* that are conceptualized virtual or socio-physical spaces for facilitating experimentation processes focused on tackling complex societal challenges by co-developing and co-testing solutions or innovations through the involvement of a diversity of stakeholders (see Almirall and Wareham 2008; Hossain et al. 2019). Under labels as *Real-World Laboratories* and *(Urban) Transition Labs*, such spaces are increasingly used as instruments for (local) sustainable transformation (e.g., Bulkeley et al. 2016; Schäpke et al. 2018; McCrory et al. 2020).

In the beginning of the project, seven Policy Labs and seven City Labs were established to, respectively, experiment with national-level policy related to food systems R&I, and work with citizens, students and other actors on city and regional levels via engagement and educational activities. In the second half of the project, 11 additional Labs (four Policy Labs and seven Food Labs^2^) were appointed, following an open call. In both rounds, organizations were selected based on their willingness to engage with transformation processes and/or their experience with stakeholder engagement. In accordance with specifications in the EC call, the project sought to achieve geographical diversity in its appointment of Labs, and to support engagement of diverse actors.

**Fig. 1:**
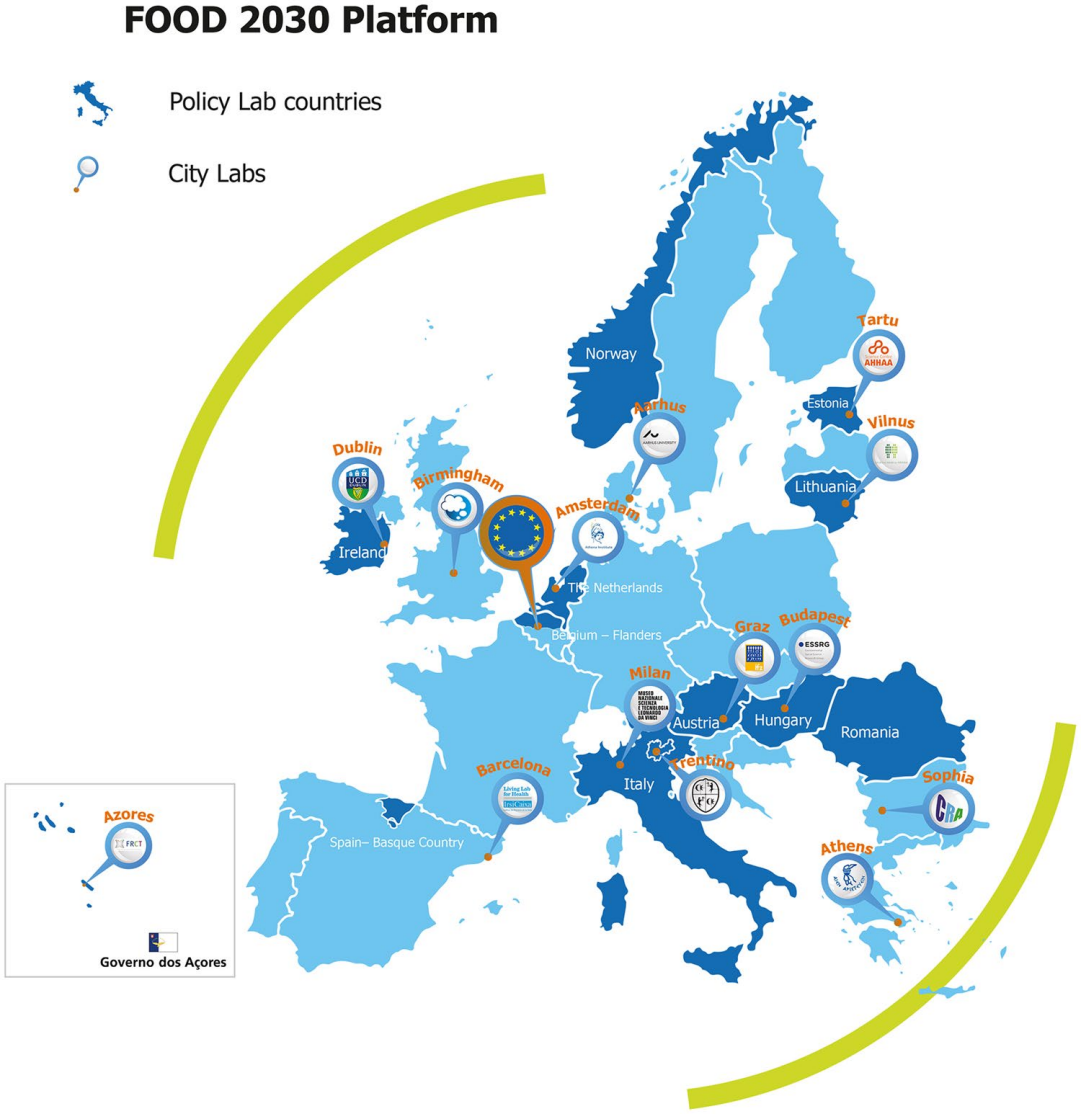
Overview of the FOOD2030 Platform and the locations of the Labs

Each Lab had one or more ‘coordinators’, responsible for the design, execution, and often the facilitation, of the Lab processes and activities. Policy Labs were coordinated mainly by employees of national ministries, while City and Food Labs were coordinated by science museums, science centers and universities. The Labs’ subsequent decisions regarding network building and stakeholder engagement were largely up to individual coordinators, informed by general guidance from the consortium regarding the desirability of including actors not usually represented in local food and R&I networks and initiatives, as well as from horizontal learning between coordinators through regular learning sessions where coordinators shared experiences and approaches (EC 2021). The consortium supported coordinators through structured discussion organized around a *Dynamic Learning Agenda* (van Mierlo et al. 2010; Svare et al. 2020a), as well as learning sessions, trainings, and materials on topics such as stakeholder diversity and engagement. Coordinators received modest project funding and a high degree of autonomy in finding synergies between content, aims and suggestions from FIT4FOOD2030, and activities, strategies, and initiatives within their host organizations and national or local contexts. An overview of Lab locations is shown in Fig. 1. An overview of Lab types, activities, and selected outcomes is shown in Table 1.

**Table 1:** Overview of the different types of Labs and their key features

Lab type	Main leverage points	Locations	Examples of experiments and outcomes
City Labs and Food Labs	Educational module co-creation (City Labs) and implementation (City Labs and Food Labs) Transformative network building	*City Labs:* Amsterdam, Athens, Barcelona, Budapest, Milan, Sofia, Tartu *Food Labs:* Aarhus, Azores, Birmingham, Dublin, Graz, Trentino, Vilnius	Local policy agenda setting, co-developing policy strategies 19 educational modules (implemented in schools, science museums, universities) engaging 1400 + students and school children Modules for instance focused on food waste reduction, systems thinking or healthy diets 1000 + stakeholders *engaged* in the Labs
Policy Labs	Policy innovations Transformative network building	Austria, Basque Country, Estonia, Flanders, Hungary, Ireland, Italy, Lithuania, the Netherlands, Norway, Romania	Co-developed R&I strategies and visions Established new transdisciplinary funding programs Cross-sectoral collaborations between governance sectors and levels 600 + stakeholders *engaged* in the Labs

The Labs were the main site of ‘doing inclusion’ in FIT4FOOD2030. They followed rigorous but context sensitive methodologies, developed or adapted^3^ by the project. These supported the Labs in four project phases:

1. **Network building and system understanding**: Labs mobilized local stakeholder networks to work on developing collective system understandings of their local food and R&I systems.
2. **Visioning and developing roadmaps**: Labs co-created visions for (the role of R&I in) future food systems and co-designed pathways and roadmaps towards sustainable futures.
3. **Action planning and experimentation**: Labs conducted different ‘transition experiments’, see examples in Table 1.
4. **Sustaining and scaling**: Labs developed and enacted strategies for sustaining their activities, networks or experiments beyond the project’s lifetime.

While it is beyond the scope of this article to report exhaustively on the differences between Labs, such differences certainly surfaced during the project, both in relation to inclusion and other topics. For example, coordinators (and their national and local contexts) differed in their familiarity with, and responses to, the project’s goals of stakeholder inclusion for the purpose of system transformation. Such differences were manifest in differences of personal experience with doing stakeholder engagement, but also emerged from differences in historical–political, geographical (North–West, Eastern, Southern Europe) and organizational contexts (universities, ministries, science museums) in which the Labs operated. These different experiences were in turn incorporated into structured dialogue and learning facilitated by the consortium. Overall, FIT4FOOD2030’s approach was one of high flexibility, aiming to be sensitive and adaptive to the local needs of the Labs, but at the same time provide a highly structured multi-phase methodology, along with the necessary training and practical tools to support the (coordinators of the) Labs.

## Research design and methodology

In this article, we present an embedded case study (see Baxter and Jack 2008), where we study the dynamics of 25 Labs as sub-units within the overarching context of the FIT4FOOD2030 project. This helps to distill lessons and findings not only within, but also across the different Labs to better unravel the ‘how and why’ of empirical dynamics (Yin 2003). During the project (2017–2020), we were involved in the management of the project (authors 1, 3 and 4), training of the Lab coordinators of the 25 Labs as well as monitoring and evaluation efforts (authors 1, 2 and 3). As the authors, we were not objective observers, but ‘immersed’ in the project and by taking an active role in fostering transformation efforts our research can be characterized as in situ* and engaging* (see Lang and Wiek 2021). Our research design was, therefore, grounded in transdisciplinary action-oriented research (Pohl and Hadorn 2007; Lang et al. 2012; Fazey et al. 2018). For researchers, to actively engage with society in action-oriented research is important *as “transformations are fundamentally about experimentation, learning, and doing something that has never been done before”* (Bradbury et al. 2019: 8). Action-oriented approaches are *“more likely to view action, learning and the generation of new knowledge as being more closely intertwined”* (Fazey et al. 2018: 58) and bring along the acknowledgment that researchers are part of the system they study; the act of research thus becoming an intervention (e.g., Fazey et al. 2018). This required us as researchers to embrace the pluralities of knowledge and values of the project partners and Lab coordinators, and to reflect upon our emerging research design and our own (multiple) roles in the project.

In these efforts, we co-designed, organized and attended (more than weekly) internal project meetings, and dozens of workshops and training sessions. In addition, the authors 1 and 2 co-conducted 28 in-depth semi-structured interviews with Lab coordinators and project partners, using a flexible interview guide, to stimulate context-specific conversations and allowing to further explore unexpected empirical insights. Questions focused on the challenges, impacts, learnings and functions of the Labs, the project and the interviewees personally. The data were selectively transcribed verbatim and coded with Atlas.ti.

Our approach to the data was an abductive one (see, e.g., Dubois and Gadde 2002) which is a style of reasoning that emphasizes theory-building through empirical observations and is a *“continuous process based on the interplay between theories and data.”* (Le Gall and Langley 2015, 38). Abduction is considered appropriate in the case of transdisciplinary action-research and semi-structured interviews (Stirling 2015), especially in the context of studying complex systems (Schlüter et al. 2019). Informed by the literature, we thus identified patterns in the empirical challenges that the Lab coordinators encountered, and discussed these together with the coordinators and project partners during the activities of the project. As researchers, we clustered the observed challenges into four major themes to construct more general conceptualizations. Data sources are summarized in Table 2. The supportive data are not used explicitly, but supplied the authors with insights into the empirical context.

**Table 2:** Details on the data used for the analysis presented in this article

Data source	Level of analysis	Function	Details
28 (online) interviews	Transcribed and coded	Main data source	15 interviews with Lab coordinators 13 interviews with core project partners involved in project coordination or Lab training
2 surveys	Coded	Main data source	Lab coordinator surveys as part of project monitoring and evaluation
Training sessions for City Lab (5 sessions), Food Lab (2) and Policy Lab (7) coordinators	Selectively transcribed and coded Participant observation	Main data source Supportive	Two-day sessions, designed in consultation with coordinators to support the Labs in addressing challenges
3 reflection sessions	Systematic field notes, coded	Main data source	3-h focus groups were organized with Policy Lab coordinators to reflect on their learnings and the impact of their Labs
21 Dynamic Learning Agenda sessions	Systematic field notes, not coded	Supportive	1–2 h (online) sessions, facilitated or observed by author 2
3 interactive webinars	1 selectively transcribed and coded 2 non-systematic field notes, not coded	Main data source Supportive	3 interactive 2-h webinars were organized. One focused on ‘power’ in stakeholder engagement, and was selectively transcribed and coded
Project meetings	Non-systematic field notes, not coded Participant observation	Supportive Supportive	Numerous project meetings, workshops, conferences and bilateral conversations
Written project materials	Not coded	Supportive	Project deliverables, publications, reports

## Analysis: unraveling political balancing acts of doing inclusion

In this section, we present four different challenges to doing inclusion in FIT4FOOD2030. While some impacts of the FIT4FOOD2030 Labs are detailed elsewhere (e.g., EC 2021), we here focus on certain patterns of challenges across different Labs, which emerged in response to ambitions for transdisciplinarity that the project sought to stimulate. Each challenge is structured around three elements: the overarching challenge, the corresponding response (or balancing act) of Lab coordinators in FIT4FOOD2030, and the implications for the politics of inclusion.

### Can we bring together the powerful and the marginalized?

#### The challenge

Bringing together both powerful and marginalized stakeholders in meaningful co-production processes was a key challenge in FIT4FOOD2030. Inclusion of established and well-connected actors or organizations could enhance the transformative capacity of Labs, for instance by providing credibility to Labs’ outcomes, and enhancing possibilities to link to ongoing transformation efforts, for instance at (local) government levels. One Policy Lab coordinator describes:

*“The involvement of these [large enterprises and government agencies] would have a greater impact and increased awareness on sustainable food systems”.*

On the other hand, inclusion of underrepresented voices broadens perspectives, increases societal support, and provides legitimacy to the process. According to a City Lab coordinator

*“The food system is rich and we want a richness of voices to understand better what they would like to embed in the activity [of the Lab].”*

Marginalized but engaged stakeholders are also important for transformation as they “*will help you more than someone who has power but not interest”* (City Lab coordinator).

#### The balancing act

In practice, balancing these (groups of) stakeholders leads to tensions. For example, a City Lab coordinator described that during a workshop a powerful stakeholder ended up at a table with clearly less powerful stakeholders. The discussion became unproductive and coordinators observed that

*“he started making comments and […] he was annoyed because there were other powerful stakeholders at other tables.”* (City Lab coordinator)

Such difficulties could be overcome by strategically designing multi-stakeholder events (see, e.g., Hendriks 2008; and more recently Pereira et al. 2018). Effective too in this regard were the project’s creative tools and methodologies (visioning exercises, co-creative pathway building exercises, see EC 2021; Baungaard et al. 2021; based on for instance van Mierlo et al. 2010; Hyysalo et al. 2019) that sought to enable equitable level playing fields in workshop settings. The effectiveness of these aids sometimes surprised Policy Lab coordinators, who observed, for instance, that high-level ministerial policy makers were happily drafting post-its and making drawings in their workshops. However, even if one strategically designs groups of stakeholders and provides appropriate tools, there is still a need for moderators to intervene in processes and discussions to ensure a certain degree of equitable participation, for instance by *“raising the level of the discussion so that the person with the weaker weight is stronger”* (City Lab coordinator).

Managing power imbalances is even more challenging when it comes to engaging stakeholders during *long-term* Lab (or transformation) processes. In general, FIT4FOOD2030 Labs reported a high degree of stakeholder diversity as well as the establishment of vibrant transformative networks (see, e.g., EC 2021). As one Policy Lab coordinator indicated: *“I can see the difference between these kind of meetings and other types of meetings that I've been to.”*

Despite the enthusiasm of those who joined the Lab activities, Lab coordinators do indicate that it was not straightforward to ensure commitment of powerful stakeholders. Some of the relevant *“policy makers [were] not very interested as generally they don’t seek feedback, but implement food related policies”* (City Lab coordinator), or even were *“afraid of the plurality and action-participatory approach [the Lab] had”* (Policy Lab coordinator). In addition, food industry sometimes did *“not see the value of such sort of activities and they may have [had] other priorities”* (Policy Lab coordinator) and farmer-organizations did not *“really see how this can be useful for them, because they're very much focused on the needs of their client”* (Policy Lab Coordinator).

Furthermore, Lab coordinators report that marginalized stakeholder groups (such as specific citizen groups, farmers or NGOs) were often difficult to continuously engage due to various reasons, including (1) the inability to convince those stakeholders that they would benefit from being included, (2) a lack of experience or legitimacy in reaching out to and meaningfully engaging these stakeholders, and (3) a lack of resources (money, time, staff) of these stakeholder groups to participate in events (see also Hendriks 2008; Turnhout et al. 2020) which could often not be compensated for by the project’s own limited financial resources. With inclusion of marginalized stakeholders also comes the responsibility to empower them:

*“The relation of trust that has to form [...] you have to be able to show that you have some power to really make a difference for the group.”* (project partner)

#### Implications for the politics of inclusion

Continuous stakeholder management is required to bring together powerful and marginalized voices both in participatory events and entire transformative processes. This also entails creating spaces for deliberation that to some degree resemble (the political dynamics of) the system but at the same time mitigate reproduction of power relations of that system. However, if this experimentation aims to contribute to transformation of the ‘system’ outside its protected space, power relations are to be restructured not only temporarily during workshops or the Lab process, but more fundamentally in the system. There lies the political challenge: to equitably include a wide variety of voices in experimenting for system transformation, is to restructure power relations of that system. Doing meaningful inclusion for transformation thus is a political intervention and relies heavily on the authority and legitimacy that process facilitators have to make decisions on how and when to include whose voices in which way.

### How do we combine representation with deliberation?

#### The challenge

A second challenge concerns the issue of speakership and representation. As we strive to classify participants in transdisciplinary processes and assess the degree and diversity of stakeholder representation, we are confronted with the challenge of how to make sense of participants’ myriad roles. When does a participant represent themselves, and when do they (also) speak for larger groups? Or more broadly: how can inclusion processes aim for diversity, representational legitimacy, or other normative ends, while accounting for the multifaceted and changing roles that participants inhabit (Maassen and Lieven 2006), to be useful, consistent, and accommodating of roles that may fluctuate over time?

#### The balancing act

FIT4FOOD2030 strove for broad and diverse inclusion as an overarching approach to food systems transformation. Trainings and guidelines designed to equip Lab coordinators with tools and approaches to organize events also stressed the importance of including relevant actors and operating with broad definitions of who should constitute the stakeholders to Lab activities, along with the encouragement of also including so-called non-usual suspects or marginalized stakeholders who did not usually have a say in food systems and related policy.

The task of operationalizing these general ambitions into something that could be carried out within the confines of Lab events (with anywhere between a handful to several dozen participants), required interpretation, selection, and prioritization, as well as choices that effectively constituted decisions about who should get to represent and speak for different participant groups. This deeply political task has significant impact on how inclusive processes unfold. It was generally carried out on the Lab-level, by Lab coordinators themselves in consultation with their core team or broader stakeholder network, rather than enacted by the project consortium or through project materials, guidelines and templates. In interviews, coordinators frequently recognized that individual participants could shift between representation roles, at one moment seeming to speak for organizations or larger groups, and at other times expressing more personal or individual views:

*“Sometimes, people participate in workshops just as themselves, with their personal interest. Sometimes just as their profession.”* (City Lab coordinator)

Often, Lab coordinators were eager to engage government authorities, together with those who could speak on behalf of groups of stakeholders as *representatives*:

*“We don't think we have a fixed network. We have a core group [...] it is not so important to have a large network. It is good to have the main authorities, and around that have a few associations, from the value chain, with industry, consumers side. And with this group we can go further with different strategies and boost the research agenda.”* (Policy Lab coordinator)

Attempts at reaching target groups via associations also proved challenging, and suggested an evolving and dynamic relation between representative and deliberation arguments for including stakeholders:

*“[I]n past years, we wanted to consult citizens through the citizens associations. But that is not really a representation of the voice of citizens. So that was not a really good way to do it. Now, we are changing our minds to use panels or groups of citizens that can be consulted on specific topics.”* (Policy Lab coordinator)

#### Implications for the politics of inclusion

The choices coordinators described above and in other interviews tended to combine pragmatic choices with normative ambitions for weighing representation and deliberation. In particular, the changing stages and topical needs emerging from Labs’ activities seemed to influence the generic ambitions to strive for engagement with large and diverse groups in the form of representation (when impact was aimed for) or deliberation (when inclusion was aimed for). Thus, coordinators reported making pragmatic and practical changes pertaining to inclusion to achieve particular goals or make certain types of progress in Labs, often opportunistically in relation to locally specific opportunities for intervening or enhancing the Labs’ impact. In doing so, coordinators had a very powerful position in ‘translating’ the meaning of deliberation and representation to their local context, and their choices strongly shaped their Lab’s direction. The implications of these observations are twofold. First, in line with the work of Hendriks (2009), it suggests that normative interpretations of democratizing participatory processes are constructed differently in different contexts and phases. Second, it suggests an intrinsic tension between inclusion and transformation ambitions in considering when and which stakeholder groups are to be engaged through deliberative or representative efforts. That brings along the political question of who decides, and with what legitimacy and authority, who is to be included in transformative processes and in which way.

### How do we balance diversity and directionality?

#### The challenge

A third challenge refers to the tricky practice of doing inclusion by balancing and fostering both directionality and diversity. As one of the City Lab coordinators illustrates, the tension is integral to complexity:

*“I think dealing with complexity means dealing with open questions that are not still resolved. Not solving conflicts, but being like an arena where people can discuss and can think about other perspectives.”*

The Labs, however, also aimed at contributing to transformation processes, and had the specific goal of experimenting with the actual implementation of one (or more) desired transformation pathways:

*“To bring people together to make a change; that is the objective. […] We did [the Policy Lab] for a purpose that served policy-making […] and in connection to the FOOD 2030 goals.”* (Policy Lab coordinator)

While there was a large degree of flexibility on the Lab level, the project already had a preset notion of creating visions and pathways within the context of transformation towards the FOOD 2030 agenda. Managing this was not straightforward, as one City Lab coordinator illustrates:

*“The food system is rich […] and sometimes, we were a bit lost in this richness. So at the end we have chosen food waste, one topic […] working on something which is very local, specific […] and on the other side something which is so wide. So, these different dimensions are not easy to manage.”*

#### The balancing act

We observed that in different situations and contexts, as well as at different stages of the Lab process, Lab coordinators (strategically) used different arguments and methods in closing down diversities and legitimized this by invoking different (democratic) values. Sometimes, decisions were reached within workshop settings through deliberation and collective decisions, as one City Lab coordinator believed that coordinators *“cannot force, because we are nobody, we are a network, we are not the owners of the network”*.

In other instances, the coordinators were more direct in steering the process in particular directions, for instance to align with the specific targets of the framework set out by the project, to make Lab outcomes more relevant, legitimate or accountable:

*“During a workshop when we were identifying clusters [...] we explained that this is the focus of our project and that the transformation needed within food production should be the focus of another multi-stakeholder ecosystem.”* (City Lab coordinator)

Directionality towards a specific thematic focus might have excluding consequences for the diversity of stakeholders. According to a City Lab coordinator, *“if we decide that we are not focusing on [food] production, it is normal that we have to ignore some of the stakeholders and incorporate new ones.”* Therefore, inclusion for transformation is in fact to balance multiple *diversities* and *directionalities*.

Interesting as well were instances where structural and socio-material configurations contributed to dynamics of inclusion and exclusion. For instance, during the Covid-19 pandemic the Labs had to re-invent themselves as virtual spaces. This allowed opening up the process for new stakeholders (for instance particular farmers, who were often not able to attend daytime Lab activities organized in cities), but led to exclusion for others (for instance stakeholders with lack of access to, or acquaintance with, digital tools and platforms).

#### Implications for the politics of inclusion

The challenge of when to intervene and on what grounds strongly relates to the different role perceptions in transformation processes (Sarkki et al. 2013; Wittmayer and Schäpke 2014). While some Lab coordinators considered themselves to be topical experts or change agents (strongly intervening in the process), others considered themselves mainly network builders or process facilitators (envisioning a more ‘neutral’ stance), while again others sought ways to combine directionality and diversity in their role-ambition:

*“I am a strong advocate for that we need an urgent and radical change in the system, however, I let go of any strong attachment with regards to how we get there. I understand now that the complexity of the issue calls for various ways and approaches simultaneously.”* (City Lab coordinator)

Our observations suggest that balancing diversities and directionalities is challenging, but that a variety of strategies and associated role perceptions can be considered (il)legitimate by Lab coordinators, stakeholders and project management. They also illuminate the deeply political role of Lab coordinators, and the powerful position they have in shaping processes (and, therefore: outcomes) of inclusion. Thus, ‘doing inclusion’ does not in itself create responsible innovation; a balancing of directionality and diversity is required throughout different phases of co-creation (van Mierlo et al. 2020).

### How are the boundaries of inclusion constructed?

#### The challenge

In the current complexity of (food) system transformation, where so many projects and experiments are initiated across governance levels, often related or overlapping, an important question arises: who is actually included in what? Consequently, how and by whom are the boundaries of inclusive experiments constructed?

In FIT4FOOD2030, we observed that this boundary-complexity affected the work of the Labs. For instance, one City Lab, contributing to setting the local policy agenda, partnered with existing networks and governments, and facilitated visioning sessions for this new network. The Lab enhanced its impact, but lost some control over who was part of the processes and activities. Another example: a Policy Lab seeking to foster collaborations between stakeholders in research, policy and society, and to co-develop funding programmes for transdisciplinary R&I, acted as a catalyst in linking existing networks and stakeholders. To increase their impact, they too partnered with existing (international) initiatives in organizing workshops and agenda-setting activities. Although one could argue that these Labs involved stakeholders from larger networks in their activities, one could also argue that to contribute to transformation, the Labs lost some autonomy over their *boundaries*. Determining and enacting the boundaries of the Labs leads to confusion on who is included in which process. It raises the question which actor, project or Lab is primarily responsible for which (inclusive) developments, and therefore, accountable for the ways in which they are shaped.

#### The balancing act

While several Labs opted for the strategy of embedding in larger institutions or partnering with (local) governments, this is not the only possible strategy, as others worried it would affect the autonomy of their Lab. One City Lab Coordinator for instance indicates that

*“when we work with the [city government] […] we have the sensation that if we were inside them, we would be collapsed by urgencies that come from the top of the [city government] […]: ‘Lab, do that now because there is a fire here!’”*

Navigating this challenge proved a complex endeavor but if coordinators managed to successfully link up to ongoing developments to create (local) impact, while at the same time remaining a degree of autonomy and flexibility to be inclusive as local spaces for experimentation, this could also be rewarding:

*“It is interesting to find on one side the balance between something which has strong priority, like the municipality, but on the other side challenge these priorities. So, swim in this big sea of policy priorities, but on the other side try to swim in an opposite direction to refresh the discussion.”* (City Lab coordinator)

Being part of a large EU-funded CSA project also brings along a role for Labs in responding and being adaptive not only to local networks and governments, but also to EU and project-level (policy) developments. This embedding of the Lab in larger policy discourses was often considered advantageous and being part of an EU-project provided the Labs with leverage to engage particular stakeholder groups, but also in their efforts to influence (local) governments, as the activities of the Lab were *“not something that we have thought of ourselves […] it’s really something that’s framed within a European project, and that’s always something that has more weight”* (Policy Lab coordinator).

#### Implications for the politics of inclusion

The boundary-challenge seemingly emerges from two paradoxical functions of transdisciplinary Labs. The first function, grounded in the desire to be inclusive, aims to create ‘Habermasian safe spaces’ to foster deliberation and reflection (see Habermas 1981; Pereira et al. 2018). To do this, one constructs boundaries to demarcate the Lab from the system, where the Lab can be an environment for co-creation and experimentation in which ‘the collective’ of stakeholders can govern itself in a democratic, inclusive and autonomous way (see also Latour’s work on the *Politics of Nature* 2004). The second function is grounded in the desire to create systemic transformation, which means that to have impact the Lab needs to open up to its environment and be adaptive to changes in the system. To scale-up its outcomes or bring into practice identified pathways, the Lab also needs to link to, or embed itself in, local governments, institutions, or existing networks that it aims to transform (e.g., Pel et al. 2020; Lam et al. 2020 on scaling mechanisms and transformative strategies).

The balancing act thus is a tricky one: inclusion requires boundary construction to ensure autonomy and inclusion, while transformation requires boundaries to be deconstructed to engage and transform the complex ‘outer world’, adding an additional layer of complexity to the already highly political nature of boundary work (Brown and Dillard 2015; Glimmerveen et al. 2020). Navigating these two critical functions simultaneously requires reflexive agency of coordinators to manage and enact multiple but, selectively permeable, boundaries of the Labs.

## Discussion and reflections: navigating the politics of transformation

As we have empirically illustrated, navigating the political dynamics in doing inclusion involves navigating multiple challenges simultaneously. Here, we present reflections relevant in the context of transdisciplinarity for transformation and point to avenues for further research.

First, the identified political challenges illuminate intrinsic tensions between efforts to combine inclusion ambitions with transformation and invigorate the notion that inclusion for transformation is as much about exclusion and ‘closing down’ as it is about ‘opening up’ (van Mierlo et al. 2020). As such, inclusion of particular stakeholders and perspectives is more relevant and justified in certain contexts and process phases than others (Schneider and Buser 2018). More in particular, we argue that balancing inclusion efforts with excluding effects they bring along forms an intrinsic political aspect of stakeholder engagement. This also raises questions on how facilitators of inclusion processes can engage in mitigating the trade-offs and dynamics of exclusion that participatory processes inevitably bring along, as well as the need to more explicitly specify which actors or institutions bear which accountabilities for which process in complexity (e.g., Glimmerveen et al. 2020) and how responsibility for and political accountability of transdisciplinary processes and outcomes, can be embedded in transdisciplinary practice and design (see also De Campos et al. 2017; Genus and Stirling 2018).

Second, we have argued that balancing this ‘opening up’ and ‘closing down’ is actually balancing multiple *‘openings’* and *‘closings’* in a number of related political challenges. *Doing inclusion* is no moment, but a constant balancing of different arguments and values; a *“political practice which is inevitably imbued with unequal power relations that need to be acknowledged but cannot be managed away”* (Turnhout et al. 2020: 18). This emphasizes the deeply political role of facilitators, as well as the power and responsibilities that come with that role in practice. Our analysis, again, indicates the importance of further exploring how (collective and collaborative) learning and building reflexive agency in practitioners involved can best take shape in transdisciplinary transformation processes (see also van Mierlo and Beers 2020; Verwoerd et al. 2020). In particular this could shed light on how the balancing of different (or even conflicting) roles between ‘action and reflection’ (Bulten et al. 2021, cp. Wittmayer and Schäpke 2014) relates to navigating the political dynamics and challenges at play in transformative efforts. Important in evaluating the legitimacy of these balancing acts is better understanding how trust building processes between stakeholders (and facilitators) take shape and how they can be further enhanced (Svare et al. 2020b), a question worthy of attention in the context of sustainable transformation (Koole 2020).

Third, our analysis implies that ‘doing inclusion’ is not only related to reflexive weighing of arguments, but requires facilitators to navigate (systemic) powering processes that result from unintended or undesirable actions and dynamics. This includes powering instigated by local (non)participants, but also project- or funder-level actions that interfere with Lab-level processes. Furthermore, though the influence of structural or socio-material powering processes is increasingly acknowledged in transition studies (see, e.g., Grin 2010; Svensson and Nikoleris 2018; West et al. 2020; Contesse et al. 2021; Kok et al. 2021), this has not yet been extensively explored in the context of inclusion in transdisciplinarity (Dannecker 2020). As we have illustrated, such structural and socio-material dynamics do, however, permeate the boundaries of ‘inclusive experiments’ and influence the dynamics of inclusion and exclusion. Further inquiries into how exactly structural and socio-material configurations interact with, or mediate, inclusivity might further enhance our understanding of why and how inclusive processes can take unexpected or undesirable turns.

Fourth, during the FIT4FOOD2030 project, Lab coordinators operating in different localities, targeting different audiences, and with different intersecting (organizational) needs, norms, and priorities were presented with normative (inclusivity, diversity) and topical (food and R&I system transformation) facilitation content. The project sought to prepare coordinators for (and stimulate learning and exploration around) challenges to inclusion. The specific ways in which coordinators faced and responded to challenges by intervening in group discussions, identifying and inviting stakeholders, or otherwise contribute so that marginalized stakeholders were not only included in formal but also substantive ways, nevertheless varied greatly. As such, it was challenging to support a highly diverse group of Lab coordinators in preparing for all the possible judgment calls and attunement to challenges they may encounter, requiring further exploration of how to best support translocal learning and empowerment processes (see, e.g., Avelino et al. 2020). Moreover, adopting more deliberate and reflexive approaches to the inherent challenges and tensions surrounding inclusion will (and should) also become reflected in the outcomes and impacts of inclusion processes—a topic outside the scope of the current article but a highly relevant focus of future research.

Finally, though we were not directly involved in ‘doing inclusion’ in the Labs, we are aware that in each of our roles (researchers, training team and project management) we were not neutral observers, but actively engaged in those contexts FIT4FOOD2030 aimed to transform. The powerful role of researchers in (agenda-setting and) shaping practice has been well documented (see, e.g., Shdaimah and Stahl 2012) and in the project, we balanced multiple sometimes conflicting roles (see Bulten et al. 2021). In fact, we were performing our own (political) balancing act: navigating between on the hand the pre-set project ambitions and targets as well as directions implied by the EU policy context and funders, and on the other hand the emergent and diverse needs of the different Labs. This required us to make difficult choices (on deadlines, stakeholder monitoring, workshop formats, etcetera) anticipating and adapting to different needs and contexts, taking both ‘project’ and ‘Lab’ perspectives in mind. Our actions too were shaped by the limited time, knowledge and resources that short-term project settings inevitably bring along. Such complexities again point to the need to enhance reflexivity, learning and capacity building not only for those ‘doing the inclusion’ on the ground, but also for those involved in supporting transformative program ambitions in a variety of different roles (see also Den Boer et al. 2021b).

## Concluding remarks

In this article, we analyzed stakeholder engagement efforts in 25 transformative Labs of the FIT4FOOD2030 project. Our contribution is threefold: first, we empirically unraveled four key challenges that emerge in the political practice of ‘doing inclusion’: (1) the challenge to meaningfully bring together powerful and marginalized stakeholders; (2) combining representation and deliberation of different stakeholder groups; (3) balancing diversities of inclusion with directionalities implied by transformative efforts; and (4) navigating the complexities of establishing boundaries of inclusion processes. Second, we explored how facilitators navigated these challenges, and emphasize that there are no blueprints or clear-cut solutions that could immediately resolve the identified challenges, as they are intrinsically embedded in the political practice of doing inclusive and transformative efforts. Third, we presented implications for the politics of inclusion, and argued that intrinsic tensions between ‘inclusion’ and ‘transformation’ ambitions pose challenges for managing transdisciplinary efforts aimed at transformation. Navigating multiple political challenges, often simultaneously, requires reflexivity, flexibility as well as rigorous methodologies at the level of facilitators, but also more broadly at the level of inclusive processes and the projects they are part of. Our findings also suggest that while focusing on concrete (transformative) outcomes is an important aspect of transdisciplinary projects, a purely functionalist take does not capture the rich and challenging political nature of doing inclusion efforts, and the potential legitimating and empowering roles that such processes bring along. Moving beyond the functional turn then also requires fostering R&I governance efforts that support transdisciplinarity through providing systemic environments in which truly reflexive transformation processes are to be enacted (Schot and Steinmueller 2018; Fazey et al. 2018, 2020; Kok et al. 2019; Klerkx and Begemann 2020; Den Boer et al. 2021a).

As we have elaborated in our discussion, our contribution also leaves many questions unanswered and requires further research along a variety of avenues. We hope that others see our contribution as an explicit invitation to engage with our findings, to further advance the understanding of how the politics of inclusion takes shape in practice. Finally, we hope that our findings can contribute to *re-politicizing* inclusion in sustainability science, and thereby to designing, *doing* and evaluating transdisciplinary processes of inclusion aimed at instigating societal transformation towards sustainable and just futures.

## Footnotes

1 Scholars agree that there are many different manifestations of the contested concepts agency and power (see, e.g., Dahl 1957; Bachrach and Baratz 1962; Foucault 1980; Giddens 1984; Emirbayer and Mische 1998; Archer 2000; VeneKlasen et al. 2002; Arts and van Tatenhove 2004; Latour 2004; Lukes 2004).

2 The project sought to move beyond an urban focus and thus labeled the additional regional Labs ‘Food Labs’ instead of City Labs.

3 Methodologies used in FIT4FOOD2030 to support multi-stakeholder experimentation in the Labs were adapted from, e.g., Transition Management (TM, see Loorbach 2007); Reflexive Monitoring in Action (RMA, see van Mierlo et al. 2010).
